# Book Reviews

**Published:** 1811-11

**Authors:** 


### CRITICAL ANALYSIS

OF

### RECENT PUBLICATIONS

IN THE

DIFFERENT BRANCHES OF PHYSIC, SURGERY, AND MEDICAL PHILOSOPHY.

---

*A Treatise on Surgical Anatomy. Part the First.* By ABRAHAM COLLES, one of the Professors of Anatomy and Surgery in the Royal College of Surgeons in Ireland, &c. &c. &c. 8vo. Dublin, 1811, pp. 219.

THE importance of anatomical knowledge to the surgeon is so obvious and so indisputable, that when practitioners are found defective in this fundamental principle, we are struck not only with regret but surprize. Anatomy, unlike abstruse and speculative inquiries, is open to the senses. It is a practical art; every thing in it may be seen, felt, and understood: therefore every student, with a certain portion of application, may as readily find his way in it, as he can from Dublin to London. But still students and practitioners are deficient in anatomical knowlege. Of this our author is fully sensible: and the object of his publication is to lay down "a plan of instruction, which should point out at each step the practical application of *anatomical researches to surgical uses.*" The utility of this plan we readily admit, because it keeps constantly within the student's view the direct application of his anatomical acquirements. Of all the motives that impel to the the acquirement of knowlege, those which point out its direct application to actual practice are the most powerful.

Two papers, "on the preparatory education necessary for the surgical student," and a "plan of study to be pursued by the surgical pupil," precede the particular business of the volume. In the first of these, which is addressed to the pupils of the Royal College of Surgeons in Ireland, Mr. Colles enforces the necessity of certain preliminary and collateral studies. Among these he ranks classical learning, and proposes that no person be admitted a registered pupil of the College of Surgeons, until he has undergone an examination in Greek and Latin. The Sciences, especially those which have been denominated natural philosophy, he also especially enforces. That part of this branch of human knowlege, which investigates the properties, principles, and relations or affinities of things, under the term chemistry, we shall particularly notice, as containing an opinion hostile to the fashion of the day.

"The advantages which the surgeon derives from the knowledge of chemistry, are not confined to the composition and administration of medicine. This science is of still more material use to him, in elucidating several important phænomena in the animal œconomy; for by chemical analysis, we acquire a more accurate knowledge of the component parts of many substances, which are secreted from the general mass of the blood, and lodged in various cavities of the body. Thus we learn more clearly the composition of urine and of bile; and thus we gain a more distinct idea of certain morbid changes which take place in these fluids, as in the formation of biliary and urinary calculi. Nor perhaps will it be deemed too sanguine a hope, to expect that chemistry may one day furnish us with remedies which shall possess the power of dissolving those concretions, aud thus free mankind from the sufferings of a most painful disease, and the necessity of a most dangerous operation. In a word, chemistry applied to the investigation of any phænomena in the animal system, which do not strictly depend on the vital principle, or employed to discover the composition of substances, which, though deposited in certain appropriated receptacles of the living body, are yet to be considered as not under the immediate influence of the living power: chemistry, I say, applied thus far, will not only facilitate researches into the animal œconomy, but may also ultimately guide our practice to a more judicious treatment. But this is the utmost extent of its utility to the healing art. Thus far, and no farther, are the principles of the one science applicable to the phænomena of the other. Here nature seems to have fixed so immoveably the common boundaries of both, that beyond those limits it appears scarcely possible for chemistry ever to extend her empire over the province of medicine. I know how contrary this is to the prevailing opinion. I know well how fashionable it is to lavish on chemistry chemistry the most unqualified praise, and to attribute to it the most unbounded utility to the study and practice of medicine; but however popular the study of this fascinating science may be; however ardent the hopes, and enthusiastic the expectations of its admirers; I trust I shall be able to satisfy ingenuous and unprejudiced minds, that the vital properties of the human system depend not on its chemical principles, and that the great and complicated operations of the animal œconomy are not subject to the same laws that govern the minute and detached particles of inanimate matter. And if I shall thereby prove the means of preventing that total disgust which will naturally be conceived to this science, on finding that time and industry had been thus thrown away, upon an attempt no less laborious than impracticable; I am confident it will be believed, that so far from being inimical to this beautiful and useful study, I am, on the contrary, strongly actuated by a sincere solicitude for the advancement of its real interests.

"To show how little the science of mutual affinities is calculated to elucidate the phænomena of animal life, we shall begin with the most simple facts, and from thence proceed to an investigation of the more complex.

"For this purpose we shall, in the first place, consider chemistry as applied merely to explain the composition and properties of the fluids, and the texture and uses of the solids. By chemical analysis then we discover, that all our fluids and solids (with the single exception of bone), are composed of nearly the same chemical principles; and that they differ from each other chiefly by having those principles combined in different proportions:—but how is it possible to suppose, that such slight differences in the proportions of the same elementary principles, can be the cause of such astonishing differences in the living properties? or indeed how is it possible, that any conceivable combination of chemical elements can impart any living property whatever? Can any chemical analysis teach us, for instance, why the elementary particles of animal matter, combined in muscle, possess the astonishing properties of motion; or how those combined in nerves communicate the still more surprizing properties of sensation? or can it explain to us, why it is, that both these extraordinary agents retain their respective powers during life, and yet lose them immediately after death, although no alteration in their component principles have taken place? If the science of chemistry then be sufficient to explain the more simple properties of any individual part, how can we sxpect it to elucidate the complicated process of any particular function? For example, can chemistry elucidate the wonderful process of digestion, by which dead vegetable matter is converted into living animal matter, and the food taken into the stomach is made to participate in the sensations of the animal whose body it nourishes? If then chemistry can neither explain the properties of individual parts, nor the process of particular functions, is it from this science we are to expect an explanation of the vital principle itself—that mysterious principle which, pervading every part of the sentient system, at once directs, directs, sustains, and harmonizes all those wonderful and complicated movements of the animal machine?

"Such are the considerations which induce me to think that the value of chemistry to the surgeon is extravagantly overrated by modern authors.

"Had these wild ideas of the perfectibility of medicine, by the aid of chemistry, remained confined to the speculations of the theorist, I should have passed them over in silence; but when I see the crude and imperfect principles of animal chemistry extensively applied to the practice of medicine, to detect the proximate causes of disease, to discover the appropriate remedies, and to explain the specific mode in which those remedies chemically operate; I feel it my bounden duty to warn you, as strongly as I can, against so dangerous a delusion. And perhaps I cannot do this more effectually, than by stating to you some few of many cases in which this attempt has been already made. The chemico-medical philosophers of the French school, a few years since, laid it down as a theory of intermittent fever, that the disease consists in a general debility of the muscular fibre, arising from the defect of gelatin in the constitution, and from the imperfect fixation of oxygen or pure air in the gelatin. From this theory it immediately followed, that the proper remedy was to make gelatin the food of the patient, and to let him enjoy the benefit of fresh air. They therefore resolved to substitute this new medicine with the modus operandi of which they conceived themselves to be perfectly acquainted, for the established specific which cured the disease, it is true, but cured it in a manner to them inexplicable. They accordingly did actually administer jelly for the cure of the ague, instead of Peruvian bark. And what was the success of the remedy? Exactly such as any man of common sense must naturally have anticipated. Thus, by the misapplication of an useful science, have these men been induced to abandon the established and successful treatment of intermittent fever, and to adopt a practice perfectly inert, founded on a theory perfectly puerile. In the same manner, and with nearly the same success, has factitious air been applied to the cure of pulmonary consumption, and oxygenated to the cure of lues venerea.

The observations of Mr. Colles on the extravagant application of chemistry to explain the causes and lay down the cure of the morbid alterations in animal bodies, perfectly accord with our own opinions. The quarrels between acids and alkalies which once shook the medical world, the notions of Silvius on the cause of fever, and the reveries of the alchymists, have hardly surpassed the wild speculations and absurd conclusions of the modern chemists. The high talents, the ardent mind, and the ceaseless inquiry of Dr. Beddoes, we cannot but reverence; but it is painful to recollect the visionary expectations which he once cherished on pneumatic chemistry. In no instance, however, have the conclusions of

 modern modern chemists been more absurd than in the hypothesis of the cause and cure of intermittents. We well remember M. Sequin's project for curing intermittent fever by dosing his patients with a solution of *carpenters glue*. According to the French statements, the success with this substance was perfectly satisfactory: and what ought to stagger our belief in medical histories, Gautieri in Italy, and Bischoff in Germany, asserted its efficacy. But where now is the hypothesis and its results? *Cinchona* still retains some character as a remedy for intermittents, notwithstanding the erudite and philosophical speculations of the chemists of France, Germany, and Italy.

That the physician and surgeon should each have a full view of the circle of medical science, would hardly require a remark, if it were not seen in actual practice that the physician is frequently ignorant of every thing that appertains to what is called a surgical case; and that the surgeon is, or affects to be, equally unacquainted with the practice of medicine, and looking no further than to the local affection. It is satisfactory to see a surgeon, who undertakes the sacred charge of instruction, representing to the student how essential it is to be acquainted with every branch of the profession.

"The talent of discriminating diseases, of distinguishing that which is before us from every other to which it may have any possible resemblance, is, of all others, the most useful to possess, and the most difficult to obtain. But it not unfrequently happens, that surgical and medical diseases mutually assume such a strong resemblance to each other, in their symptoms and characters, that it becomes a matter of serious difficulty to discriminate between them. In such diseases then, the practitioner cannot possibly ascertain under which of them his patient labours, unless he be perfectly acquainted with the characters of both. For instance, if a surgeon, unacquainted with medical disease, were called to visit a patient affected with a recent inflammation of the testicle, he would, in all probability, at once apply those topical and general remedies which the rules of surgery direct; instead of first ascertaining whether the disease had been preceded by a degree of fever, and a swelling of the parotid gland. And thus, by mistaking a mere consequence of cynanche parotidea for an original disease of the part affected, he might seriously endanger both his own character and the life of his patient.

"But medical knowlege is no less useful to the surgeon in the treatment than in the discrimination of diseases. For it often happens, that a patient labouring under a surgical complaint, is attacked with a medical disease, which though not originally connected with the local injury, may yet act on it in such a manner, as to produce very material changes in the symptoms. Under these new and alarming appearances, the surgeon, if ignorant of the origin and nature of the the constitutional complaint, would be led to adopt a treatment for the surgical disease, unnecessarily severe or absolutely dangerous. Thus a patient labouring under a wound of the scalp, may be seized with idiopathic fever; and this disease may produce alterations in the wound, resembling those which take place when the parts within the skull are engaged in the injury. Were the surgeon, under these circumstances, ignorant of the causes of ordinary fever, he might rashly proceed to the operation of the trepan. It may, however, be urged, that the surgeon will find it most prudent, in all medical diseases, to call in the aid of a physician; but to this there exists an insuperable objection, namely, that in all dubious and difficult cases of mixed disease, an ignorance of surgical diseases must incapacitate the mere physician, just as much as an ignorance of medical diseases can incapacitate the mere surgeon. If a physician be called to treat the fever which often attends strictures of the urethra, he might conclude, from the similarity of the symptoms, that his patient was attacked with intermittent fever, and would accordingly pour in bark and other remedies, calculated for the cure of that disease, by which the symptoms of the fever would be rather exasperated than relieved."

From these preliminary observations, the author proceeds to give the anatomy of the inguinal, congenital, femoral, and umbilical herniæ; the anatomy of the abdomen, thorax, neck, throat, and pelvis; with the organs of generation, rectum, perinæum, and bladder. To which is subjoined a chapter on passing the catheter, and another on the operation of lithotomy.

In an elementary work, intended for the use and instruction of the student, we do not look for original materials, profound phisiological research, or new views of science: if what is already known, is given with clear arrangement and in perspicuous language; condensed sufficiently to bring it under the eye, but enough dilated to leave nothing obscure, the intention is fulfilled. If in the execution of his design, Mr. Colles has not perfectly succeeded, he has produced a treatise that will be useful to the tyro. If his language has not now that elegant precision, which habit in composition only can give, we may hope that as his work proceeds, for this purports to be a first part, that practice will give polish to his style, and further contemplation of his subject induce greater clearness of arrangement. Of its importance we feel the same sentiment with Mr. Colles; and we agree with him, that "*among all those sciences which are subservient to the practice of surgery, anatomy justly challenges the first and highest rank: it is not only of the greatest importance, but of indispensible necessity, both to the study and practice of the surgeon.*"

### *Thomson's London Dispensatory.*

(Concluded from page 337.)

In our preceding number having given some account of the first and second parts of this work, and subjoined the article on Cinchona as a specimen of the author's design and a favourable instance of its execution, we now proceed to notice the third and concluding part.

In this part, under the title of "Preparations and Compounds," are described the compositions of the London, Edinburgh, and Dublin Pharmacopœias. These are arranged in the following classes:—Acids, alkalies, and neutral salts—earths, and earthy salts—preparations of sulphur, —metallic preparations--preparations of vegetables, expressed oils, volatile oils, distilled waters, decoctions, infusions, mucilages, extracts, mixtures, spirits, tinctures—preparations of æther—wines, preparations of vinegar, preparations of honey, syrups, confections, powders, pills, troches—preparations from animals, plasters, cerates, ointments, liniments, and cataplasms. To this is appended a history of common and mineral waters, the method of analysing the latter, and a useful table containing the synonyms of the Lond. Edin. and Dub. Pharmacopœias, with the doses of the various articles.

From this part we shall extract an original article on water, inasmuch so as it has not been thus treated of in Dispensatories, though its importance in the operations of nature, its efficacy in the cure of diseases, its supposed agency in the production of morbid effects, and the share it has in pharmaceutical operations entitle it to be distinguished among the bodies that constitute our Materia Medica.

"The appearance of common water is too well known to require description. It retains its fluidity under the ordinary pressure of the atmosphere, at any degree of temperature between 32° and 212°; but under 32° it crystallizes and becomes solid, and above 212° assumes an aëriform character, expanding to 2000 times its ordinary bulk. One cubic inch of pure water at 60° and under a pressure of the atmosphere, indicated by 30° of the barometer, weighs 252.422 grains, or $\frac{1}{11}$ of a grain less than 252$\frac{1}{2}$ grains.

"Although water is almost universally diffused over the surface of the earth, yet it is not found perfectly pure in any place; which is owing to its great solvent powers enabling it to take up a portion of many things with which it must come in contact in its natural state. These impregnations, however, are not sufficient, in general, to give it any sensible taste or odour, or to render it unfit for the ordinary purposes of life; and it is in this state that ordinary nary water is usually obtained. Common water varies considerably according to the source whence it is derived, and other circumstances; but all varieties may be reduced under the following heads:

1. Rain water—*Aqua pluvialis.*

2. Spring water—*Aqua fontana.*

3. River water—*Aqua pluviatilis.*

"RAIN WATER is the purest of natural water: but it, nevertheless, contains in solution, in every 300 cubic inches, about $3\frac{1}{2}$ cubic inches of air, rather more oxygenous than common atmospherical air, and about one cubic inch of carbonic acid gas, beside minute portions of carbonate and muriate of lime. Its specific gravity scarcely differs from that of distilled water, and after precipitating the muriate of lime, by dropping into it a little barytic water, and exposing it to the atmosphere until the precipitate be totally deposited, it is sufficiently pure for most pharmaceutical purposes. (Morveau, *Annales de Chimie*, xxiv. 320.) When rain water, however, is collected in towns, or from the roofs of houses, it contains a small portion of sulphate of lime, soot, and other impurities, and requires to be boiled and filtered be fore dropping in the barytic water.

"*Snow water*, when newly melted, is destitute of air; and when allowed to remain for some time exposed to the atmosphere, it does not differ in its qualities from rain water.

"2. SPRING WATER, if it has not been filtered through a very soluble soil, is almost as pure as rain water. The best springs are those which rise through sand or gravel, at a small depth.* It generally contains, besides the above mentioned ingredients, a small portion of muriate of soda.

"*Well or pump water*, which is spring water obtained by digging to a considerable depth, is by no means so pure. It is commonly distinguished by a property named hardness, implying an incapability of dissolving soap;† which is owing to its containing many earthy salts, the principal of which is sulphate of lime. Many of the foreign ingredients contained in hard water are simply suspended in it; for pump water is rendered softer and purer by only passing it through a filtering stone. The best mode of freeing hard water of its earthy salts, is first to boil it; then, after it has cooled, to drop into it an alkaline carbonate; and lastly, to filter it. It cannot be employed for pharmaceutical purposes.

"3. RIVER WATER, when the stream is rapid, and runs over a pebbly or siliceous channel, is as pure as the softer spring water; but when the current is slow, and the bed clayey, it approaches nearer to the nature of well water, and frequently contains putre-

* The water conveyed to Hoddesdon, in Hertfordshire, rises through a fine white sand, and is so pure, that Dr. Hales affirms, it left no incrustation in a boiler which had been in constant use for fifteen years. *Statical Essays*, 2. 242.

† Soap when agitated with hard water is decomposed; the alkali of the soap uniting with the acid of the earthy salts, while the oil and earths combine, and form new nearly insoluble soaps, which swim in a curdy form on the surface of the water.

fied fied vegetable and animal matters, as is generally the case in water of lakes and marshes.

"Such are the foreign ingredients contained in *common water*. Boiling frees it from air and gases, and precipitates many of the earthy salts; but distillation in glass vessels frees it entirely from them, and it is obtained almost perfectly pure, transparent, colourless, insipid, and inodorous.

"The varieties of water enumerated above may be almost indiscriminately employed as diluents, the small proportion of foreign ingredients they contain, occasioning no difference in their diluent properties. When the quantity of sulphate of lime and aluminous matter, however, is ever considerable, as is the case in many pump waters, there is some reason for concluding that deleterious effects may arise from the use of the water; although it may be doubted whether the scrophulous and glandular swellings, peculiar to some populous towns, can be justly ascribed to this cause.* Even a few of the waters which are regarded as mineral waters owe more to the diluent property of the water for their efficacy, than to the impregnations they contain. This is particularly the case with the Malvern spring, which has been found to contain very little foreign matter. The diluting power of water is much modified by temperature; warm or tepid water being a much better diluent than cold water.

"The medicinal properties of water as a diluent were well known to the ancients; and cold water used as a drink in fevers, was the principal remedy of the father of physic in these complaints. The temperature of 60° is the proper degree, when it is intended that water should produce its diluent effects without the aid of heat. Under 45° it produces a sedative and astringent effect; above 60° and under 100°, it relaxes the fibres of the stomach, and is apt to induce nausea, particularly when bulk is added to the range of temperature; but at a higher temperature the stimulus of heat, in the same manner as the addition of other stimulants, prevents that effect. Simple water may supersede the use of all other diluents; but animal and vegetable infusions are generally employed, or toast and water (*infusum panis tosti,*) which is more agreeable to most palates, and is an excellent remedy in fevers and inflammatory diseases. The temperature of water as a diluent should be regulated by the nature of the disease: in internal hæmorrhages the temperature should not exceed 45°, but it may be 60° in fevers; unless in the cold stage of the paroxysm of fever when thirst should be allayed by tepid or warm water, or other bland fluids; and the same precaution is necessary when the sweat has become general and profuse. In cases in which there exists a morbid increase of bile disturbing the functions of the stomach and irritating the bowels, the temperature of the water used as drink may be from 90° to 114°;

---

* Percival ascribes the glandular swellings common in Manchester to this cause. See *Essays*, I. 291.

and and in some cases of dyspepsia, which are attended with the sensation of coldness at the stomach, and with cold extremities, a cupful of water, taken as hot as it can be drank, affords very considerable relief. In cases of redundant bile, by drinking half a pint of tepid water every morning before breakfast, and taking immediately afterwards moderate exercise, the acrid bile is diluted, and its passage through the bowels assisted, without the irritation, which in its undiluted state it always excites; and it produces the same benefit in cholera morbus in the commencement of the disease, the stomach being rendered by it more fit to receive opiates and other remedies. Some medicines, as sudorifics, diuretics, and emetics, scarcely produce their effects, unless their operation be assisted by copious dilution with water, or watery fluids.

"Water is also an external remedy of great importance, but its effects are much modified by the degree of temperature at which it is applied.

"Cold water, or of a temperature under 70°, gives the sensation of cold to the skin, and is applied under the form of *bath* and of *affusion.*

"The cold bath, when the body is immersed in it, first induces the sensation of cold, excites shivering, renders the skin pale and contracted, so as to produce the papillous appearance denominated goose skin *(cutis anserina)*; the respiration at the same time is quickened and rendered irregular, producing sobbing; and the pulse is diminished in force and velocity, but is also rendered firmer and more regular. If the immersion be not long continued, reaction takes place on coming out of the bath, a glow, or agreeable sensation of heat is felt over the whole body, the tone and vigour of the muscles are increased, a buoyancy of spirit and aptitude for action succeed, and a sense of general refreshment is experienced by the bather. The protraction, however, of the immersion for a considerable space of time, particularly if the temperature of the bath be under 50°, is not followed by this reaction, but the cold water operates as a powerful sedative; the action of the heart and arteries becomes languid, the pulse ceases at the wrist, the animal heat is rapidly diminished, and a sensation of coldness at the stomach is felt, which is succeeded by faintness, delirium, torpor, and death. Sometimes these unpleasant effects are experienced in some degree, even when the immersion is not protracted, and the temperature of the bath is not under 60°; in which case cold bathing proves always hurtful, and ought not to be repeated; but when the contrary effects are experienced, it is found to be useful in many diseases of debility, particularly in scrophula, if the water be impregnated with salt; or see bathing be resorted to. The use of cold water as a general bath is never employed with a view of producing its sedative effects; but for this purpose is partially applied either by the immersion of the affected parts, or by means of cloths dipped in very cold water, laid over or near the parts. It is used as a remedy in uterine active hæmorrhages, burns and scalds, and in local inflammations, even when arising from general disease, as gout and acute rheumatism, rheumatism, when the surface of the pained part appears red and inflamed.

The cold effusion, or the suddenly pouring cold water over the whole surface of the body, operates as a powerful stimulant, although its effects are of short duration. They are produced by the suddenness of the application affecting the nervous energy, and by the shock rousing the dormant susceptibility, so as to induce a new action, as it were, of the nervous system, dissolving the spasms on the extreme vessels on the surface, carrying off a large portion of morbid heat by general evaporation, and the remainder by insensible perspiration; thence restoring the healthy action of the exhalents and the capillaries. In typhus fever, this mode of applying cold water has been productive of the best effects It should be applied in the first hot stage of the disease, if possible, and repeated every time the morbid heat returns. If the water can be impregnated with salt, so much the better; but when the disease is advanced, its temperature should not be more than 26° under the heat of the body. It often stops suddenly the disease, if it be used during the three first days, and sometimes so late even as the fifth; but after this period, it can be regarded as an useful auxiliary only, when properly used. In tetanus, Currie affirms (*Reports on cold Water*, i. 138.) that the cold affusion also proves useful, particularly when the shock is considerable, and applied during the presence of the convulsions. It is, however, in idiopathic tetanus only that it proves useful, no advantage being obtained from using it in tetanus arising from wounds. Its utility has also been proved in many of the exanthematæ; for instance, during the hot stage of the eruptive fever of small-pox; and we can bear ample testimony to its efficacy in scarlatina maligna, when the heat rises to above 100°.* This remedy, however, is productive of much mischief when misapplied; and therefore it is necessary to observe that it is contraindicated in the cold stage of fever, and when a sense of chilliness is present, although the thermometer indicate the real heat to be more than natural. It is also improper in fevers, when diarrhœa or dysentery are present; after the sweating stage of intermittents is performed; after the eruption is completely formed in confluent small-pox; and in symptomatic fever occasioned by great local inflammation. The cold affusion, in the form of the shower bath, is advantageously employed as a stimulant and tonic in diseases of general debility, when no fever or increased heat is present.

Warm Water, or of a temperature from 70° to 100°, gives the sensation of warmth to the body, and is applied both locally and generally in the form of vapour, fomentation, and bath. Water is found in

* Currie gives the following results of the affusion:—The heat of the body in fever, as indicated by the thermometer, being 103°, was by it reduced to 98° in half an hour, and the pulse from 112 to 80 beats, (Vol. i. 22.)—the heat 101 was reduced to 99, and the pulse from 112 to 98 in the same time. The heat 106 was reduced to 98; and the pulse from 130 to 90. (Vol. i. 46.)

a state a state of nature, combined with different quantities of caloric, within the above range of temperature. In the Buxton hot-springs the temperature is about 82° ; at Bristol it is from 76° to 84° ; and at Bath the range is from 110° to 114°. The necessary degree of temperature, however, is generally observed by artificially heating the water.

"The general application of warm water is obtained by means of baths. When the greater part of the entire body is immersed, the water constitutes properly a bath *(balneum)* ; but when half only is immersed, it is a half bath *(semicupium)*. These may be either a. The hot bath *(balneum calidum,)* from 97° to 100°.

b. The tepid bath *(balneum tepidum,)* from 62° to 90°.

c. The vapour bath *(balneum vaporis.)*

"The two first differ in temperature only ; but the last, from the water being applied in a very minutely divided state, acts with much greater effect than water in a liquid form. The operation of the first of these forms of applying water is stimulant ; it augments the action of the heart and arteries, renders the skin red, quickens respiration, and produces a copious flow of sweat; but the others, although they excite the sensation of heat, yet lessen the frequency of the pulse, relax powerfully the skin and simple solids, and diminish, generally, increased excitement. It has been a question of some interest, whether water be ever taken into the body by the cutaneous absorbents ? That it is taken in has been denied by many philosophers, and facts brought forward to support the opinion. Dr. Currie and Dr. Pearson, after half an hour's immersion into the Buxton bath, at 82°, found that the weight of the body was rather diminished than increased ; and in a case of disphagia, in which neither food nor drink was taken by the mouth for a considerable time, the patient when put into a tepid bath felt his thirst alleviated, and received much comfort, without his weight being at all increased. Dr. Currie supposed that the abatement of thirst in this case arose from the relaxation of the exhalents of the surface produced by the bath, and those of the stomach sympathizing : and that although the exhalents terminate by open mouths which pierce the epidermis, yet as the mouths of the absorbents terminate under it, and do not come into contact with the open air ; so while the epidermis remains unirritated and entire no absorption of fluid can therefore take place from the surface. Many experiments made by Seguin are also in favour of the opinion that no cutaneous absorption is effected in the bath. Among others, he immersed venereal patients in baths containing oxymuriatic of mercury in solution, and found that while the cuticle remained entire, no salivation, nor other effect of the mercury on the system, was apparent ; but the specific effect of the remedy always took place when the epidermis was injured or destroyed, as in itch. It must, however, be observed, that in the case of disphagia the urine flowed as if drink had been taken by the mouth ; a circumstance which Currie supposes to depend on the absorption from the atmosphere by the lungs. This, however, is an assumed position : the free exhalation from the lungs is evident, but it is by no means proved that any absorption takes place. It is true that the weight of the body in the above case was diminished ; but from the sum of this loss we must abstract the cutaneous exhalation of the part of the body not immersed, the pulmonary exhalation, and the weight of the egesta : and and were a supposition to be admitted as argument, it might be suggested that the relaxant power of the warm water acting on the epidermis as on inert matter, may open a way through it to the mouths of the absorbents. The question is still undecided, and fortunately it is not of much importance in a practical point of view.

Warm and vapour baths are efficaciously employed in acute rheumatism, inflammation of the abdominal viscera, of the kidneys, bladder, and uterus, in suppression of uterine, and in spasmodic affections, particularly those to which infants are liable, arising from dentition and other irritations. The general relaxation produced by their use has been taken advantage of for assisting the reduction of strangulated hernia ; for, although the effect be not topical as it regards the hernial tumour, yet the general relaxation produced gives a disposition to all the parts to regain their proper place. The tepid bath is found to be useful in the rigidities which follow some acute diseases, as gout and rheumatism, in nodosities of the joints ;* and according to some, the rigidities attendant on old age.† Its effects in promoting the natural excretions by the skin, render it very serviceable in promoting the cure of herpetic eruption ; in slight cases of lepra the use of it with friction is all that is required ; and in all cutaneous foulnesses it is a most important auxiliary.

"The partial application of warm water as a remedy, is made by means of

1\. a. The foot bath (*pediluvium.*)
b. The hip bath (*coxæluvium.*) And
c. The hand bath (*manuluvium.*)
2\. d. Fomentations of vegetable decoctions : And
e. Flannel cloths wrung out of boiling water, by which the moisture is applied in a state of vapour.

"These partial baths are useful in the same diseases for which the general baths are employed ; but are better adapted for relieving the rigidity of single joints, and topical inflammation ; and the hip bath has lately been found to be very beneficial in suppressed menstruation, and for relieving the pains of cancer in utero.

"For fomentations it is the practice to employ vegetable decoctions, but the best of these can be regarded only as vehicles for retaining the heat and moisture. At all times, flannel cloths, wrung out of boiling water, are superior: both because the water is applied in the form of vapour, and also while they continue as long warm, they do not wet the bed and linen of the patient. The flannel cloths should be each about two yards long, with the ends sewed together ; so that by means of two sticks, one being at each end, turned in opposite directions, they may be wrung much dryer, when taken

---

* Haygarth, *Clinical History of Diseases.* 8vo. Lond. 1805.

† Tepid bathing with friction is said by one author, "vitam sæpe per plures menses, interdum etiam per aliquot annos, protraxisse." Gregory, *Conspectus Med.* ij. 100.

out out of the boiling water, than could be effected by the hands. The principal circumstance to be attended to in the application of fomentations, is the frequent renewal of them, in order that a steady and constant heat may be applied to the fomented part."

Although we admit, and we do it very readily, that this London Dispensatory has considerable merit, we cannot but observe that a history of the Materia Medica is not a mere commentary on this or that Pharmacopœia; it is a detailed view of whatever the store-house of Nature contains for the preservation of health or the cure of disease. After the most laborious compiler has gone over the London, Edinburgh, and Dublin Pharmacopœias, and all the continental dispensat[illegible]ies, he will have left many medicinal substances untouched. Valuable as these works may be, they have been influenced by the fashion of the day, and are always published under the partialities or prejudices of particular schools. Nations of high antiquity, as Hindoostan; partially civilized people; the untutored savage; and the mass of European population, have among them many substances, rejected by these official volumes, though known to afford efficacious remedies, and which claim a place in the records of science. We should have been better pleased if this gentleman, who appears well qualified, had undertaken a history of the Materia Medica on this broad basis. We also believe it would have given him a higher station in the scale of medical literature. From his present work he must be content to derive no more than the character of an imitator. He may have gone a few steps further than his prototype; but as his arrangement and design are nearly the same, his work can be considered as little more than an improved edition of the Edinburgh Dispensatory. Was, however, such a volume wanted? The booksellers' shops are full of them. But there is an hiatus in this branch of medical literature, which Mr. Thomson might well have filled up. A work on the Materia Medica, elaborate in its research, classically perspicuous in its composition, comprehending the knowledge of all periods and places both scientific and popular, on this subject, made, not for the dispensor's shop but for the physician's library, is yet a desideratum. To the Materia Medica this book might be what Burney's History is to music.

If we mistake not, Mr. Thomson has feelings above a mercenary compiler; and as we are not disposed to doubt his powers, we recommend such a plan to his notice: yet, as this plan will involve laborious research, and no inconsiderable expence, we cannot expect to see it executed, except by some person who unites an attachment to the subject with extensive acquirements; and, above all, an ardent desire for reputation.

"Fame is the spur that the clear spirit doth raise,
(That last infirmity of noble minds),
To scorn delights and live laborious days."

---

*Journal Génerale de Medicine, de Chirurgie et de Pharmacie; ou, Recueil periodique de la Société de Médecine de Paris;* Redigé par M. Sedillot (In), D. M. &c. &c. Tom. XL. Paris.—No. 173.—Janvier, 1814.

As many of our readers, especially those who reside in the country, can have few opportunities of perusing foreign journals, we purpose to present an analysis of the articles (in such of them as we can obtain) which possess sufficient interest.

The first number of the volume before us opens with a memoir on the different modes of treating tetanos in America, preceded by an account of the good effects of the fruit of *Solanum carolinense*, and the juice of the garlic (*Allium sativum*), in that disease, by Dr. Louis Valentin.

In the first case, the patient was affected with *emprosthotonos*; the spasms were general and frequent, the lower jaw pressed firmly against the superior, and deglutition was extremely painful. After trying various remedies, stimulating, emetic, cathartic, &c. for three days, they were discontinued, and Dr. Valentin prescribed an infusion of the fruit of *solanum carolinense*. The potion was repeated every evening, the strength of the infusion being augmented, with the effect of procuring sleep and perspiration, relaxing the spasms and stiffness, and in a few days the patient was entirely restored.

The author professes to have only tried this remedy in two cases, and admits that doubts may arise respecting the cure of the disease being fairly attributable to its use.

The garlic was applied by friction along the spine and extremities, and the juice was at the same time taken, in the dose of a spoonful two or three times a day. The friction produced painful irritation, with redness of the skin, and the spasms yielded. In some instances this remedy proved anthelmintic. It must be observed, that none of the cases of tetanos, treated in this manner, were the severe forms of the disease which sometimes are occasioned by wounds, and are seldom cured.

The author proceeds to the treatment usually employed in America, where punctures, and even slight wounds, particularly in the extremities, frequently occasion tetanos.

During

During the civil war in St. Domingo, he mentions that they had numerous cases of the disease, and most of them from gun-shot wounds; they generally were fatal; he does not recollect a single case being cured in the hospital.

The treatment which proved most successful, consisted in applying, immediately after the accident, emollient cataplasms, and embrocations, with oil of olives or almonds; bathing the fingers, hands, or feet in it, if there was laceration of the tendonous sheaths, or aponeurotic membranes. Sometimes it was necessary to add opium to the embrocations.

As soon as the first symptoms of the tetanic affection appears, if it is a contused wound, the author directs an incision to be made; if a punctured wound, incision and cautery with red hot iron.

He remarks, that it is very common to see negroes attacked with tetanos from the prick of a nervous filament, by a very slight wound made by a nail in the feet or toe, sometimes without the cause being apparent. Upon attentively examining the extremities in these cases, he sometimes only discovered a slight contusion, covered with a scab: upon making an incision, and exciting suppuration by sinapisms or cantharides, the convulsive motion and stiffness gradually yielded.

A negro, after having the sole of the foot (very near the little toe) punctured by a nail, had been affected for two or three days with locked jaw and clonic spasms. The mark where the nail entered was hardly perceptible. He was cured by the local application of hot iron, sinapisms, anodyne embrocations, and pumping sea-water over the head and whole body. It is remarkable, that this man having refused the introduction of nourishment which was proposed, by means of an elastic gum catheter up the nostrils, took no food or medicine internally for more than seven days.

The author mentions, that the son of Blanchard, the aëronaut, died suddenly of tetanos, at New York. A shed, in which were placed a balloon and apparatus necessary for an ascension, being struck by a thunder storm, fell; in attempting to extricate them, a nail entered into the heel of the unfortunate youth. Incision and the actual cautery, for which nothing could be substituted, were neglected.

Numerous facts, he continues, prove the importance of topical treatment in such cases. In some instances it should be applied immediately after the accident: for want of this precaution, Edward Wyer, a physician, at Boston, sunk under tetanos; a nail had perforated the sheath of the flexor tendons of the second toe; as some drops of blood flowed from from the wound, he contented himself with applying a little oil of petroleum. The third day he was able to pursue his professional avocations;—on the thirteenth, the first symptoms of tetanos declared themselves by contraction of the muscles of the neck, and difficulty in swallowing. Notwithstanding the employment of various remedies, and the constant care of his friends, he died on the twenty-second day of the puncture, and the tenth of the disease.*

The author found a solution of opium united to emollient lotions, and in some cases alone, of use in dressing the wound; also embrocations of camphor, oil, and opium round it; and along the spine, and the inner part of the limbs. Cataplasms with opium were serviceable: and in some instances excision of a part, and amputation of a finger or injured member, prevented or removed tetanus.

The case of Johnson, a young Virginian, was thus treated. Firing off a gun it burst in his hand, which was much torn, and the flexor and extensor tendons were laid bare. On the first indication of constriction of the throat and jaws, Dr. Valentin, fearing the consequences, obtained permission to amputate the hand. The patient took a few grains of calomel and opium and was cured.

Several other successful cases, in which amputation was performed, are selected; and some fatal ones in which it was omitted.

The author mentions that opium is very freely used in America in tetanic affections; Dr. Chalmers of Charlestown gave an ounce of laudanum in twenty-four hours; Dr. Gloster, of Antigua, gave fifteen hundred grains of opium in seventeen days; Dr. Dazille, who practised a long time between the tropics, especially at St. Domingo, only found it useful when several grains were taken at once. From half a dram to a dram of solid opium may be given to adults with safety every three or four hours. The only inconveniences which have resulted in these cases have been constipation, scanty urine, dysury, or strangury. But sometimes these symptoms are peculiar to the disease. To obviate costiveness purgative glysters were used.

Dr. Blane, our author observes, has stated that opium and the warm bath proved the most efficacious in cases of tetanos from wounds after a sea-fight. After the battle in April 1782, he observes, 354 of the wounded died, and of these sixteen were carried off by *trismus*.

---

* The particulars of this interesting case are detailed in Memoirs of the Academy of Arts and Sciences of Cambridge (New England), Vol. II. Pt. I. p. 192.

Dr.

Dr. Petit, of Lyon, out of fourteen cases of traumatic tetanos, only attributed two cures to opium combined with aq. ammonia acetat. theriaca, and oily embrocations. All those which proved fatal, he says, were treated with opium, camphor, vermifuges, bark, blisters, baths.*

The author cites various authorities in support of the benefit derived from opium, several of them English; he concludes this part of the subject with considering the effects of cold baths and cold affusion. The result seems to be in favour of the application.

(*To be continued.*)

---

*Recherches de Physiologie et de Chimie pathologiques, pour faire suite à celles de Bichat sur la vie et la mort. Par* P. H. Nysten, M. D.†

This work is divided into five sections. In the first, the author determines the effects produced upon the animal œconomy by the presence of gas in the sanguineous system. In the second, he considers the chemical phenomena of respiration in diseases. The third treats of the changes in the secretion of urine. The fourth contains an examination of the vital properties after the extinction of general life. In the fifth, the author inquires into the stiffness which bodies contract some time after death.

Several eminent authors, as Ruysch, Morgagni, Haller, Lieutaud, &c. have remarked that upon puncturing the blood-vessels, in the corpse of certain individuals, bubbles of air are disengaged. M. Nysten, and with him several respectable physicians, have recently observed the same phenomenon.

It has hence been supposed that death might be attributed to the presence of these air-bubbles in the sanguineous system; even about the middle of the seventeenth century this simple conjecture seemed to be converted into a demonstrated truth, in consequence of the experiments of Redi, and a number of physiologists, who had witnessed the injection of a certain quantity of gas in the venous system, followed by the death of the animals submitted to the experiments. Even Fontana and Bichat believed that a very small portion of gas would suffice to produce fatal effects.

---

* Discours sur les maladies observées pendant neuf annés à l'Hotel-Dieu de Lyon. Essai sur la médicine du cœur.

† (Journ. Gen. de Med.)

some

Some experiments, however, having given M. Nysten results very different from these opinions, he was convinced,

1st. That of thirteen gases which he has examined, there is not one which may not be injected in the sanguineous system, in small quantity, even of those which are termed deleterious, such as nitrous gas, sulphureted hydrogen, ammoniacal gas, and oxigenated muriatic acid.

2. That the non-deleterious gases, injected in the sanguineous system, produce their consequences by acting mechanically on the organs of circulation; an action in the inverse ratio of the solubility of the gas.

3. That, on the contrary, the deleterious gases act by their chemical properties; thus, ammoniacal gas and oxigenated muriatic acid, by producing a sensible irritation on the organs; nitrous gas by changing the condition of the blood; lastly, sulphureted hydrogen by especially affecting the cerebral nervous system, and quickly destroying its sensibility.

In his researches into the chemical phenomena of respiration in patients, M. Nysten used the machine of Girtanner, which he simplified by some slight modifications. The following are the results of a certain number of experiments conducted by this apparatus.

1. In chronic diseases, without fever, and without lesion of the organs of respiration, the air expired is nearly the same as in the healthy state.

2. Acute severe fevers sometimes appear to occasion an increase of carbonic acid.

3. When there is considerable obstruction in the respiratory organs, the air expired contains less carbonic acid than in the ordinary state.

4. If the same air used in the experiment is breathed a long time, it will be found to contain an augmentation of azote.

5. The residue of pure oxigen, and that of hydrogen respired in the same manner, present a notable quantity of azote and carbonic acid.

6. The product of the respiration of carbonic acid gas also contains azote, and the residue of the respiration of azote includes a certain quantity of carbonic acid; but, in this case, the azote, far from augmenting, diminishes.

Although most of these facts have already been ascertained and disclosed by Messieurs Bertholet, Allen, Pepys, and others, the researches of M. Nysten are nevertheless interesting and important, and it is to be desired that he will continue his investigation on the subject.

Upon the alterations in the urinary secretion, the author gives an exposition of the analysis which he has made of the urine in nervous, inflammatory, and dropsical complaints. The

The urine in nervous cases resembled that voided after drinking; in inflammatory cases urea, saline substances, and albumen abounded; the turbid scanty urine in dropsy is ammoniacal; contains acetic acid, sulphates, alkaline muriates and phosphates, coloring matter, and albumen, but urea is not evident.

M. Nysten then treats of the deviation of urine; after quoting some observations from the ancients on this singular affection, he relates two facts which occurred to his notice, one in a young woman aged 26 years; the other in a woman aged 40. According to the author, these females voided their urine by vomiting. Upon the authenticity of the first of these cases we cannot decide, but the second was an imposture; for since M. Nysten's publication it has been ascertained, that there was no deviation of the urine, no fœcal matter, no menstrual flux, discharged from the mouth, as affirmed by the pretended patient. It seems that this miserable creature had swallowed the matters in question, unknown to the attendants; and being watched, and not allowed the use of her hands, the cheat was discovered. The French editor remarks that it is not so astonishing that M. Nysten, and several other French and foreign physicians, who only paid transient visits to this woman, should be duped; but he very justly observes, that it was indeed remarkable that the learned Professor to whose care she was confided, and numerous pupils who daily examined her, should suffer themselves to be imposed upon so grossly for upwards of ten months. This example, he continues, shews the reliance we should place on most of those extraordinary facts which certain authors are so fond of communicating, and warns us at the same time to be very circumspect, whenever we have occasion to decide upon a fact which seems to derogate from the usual laws of nature.

Most of the experiments relating to the vital properties examined after the extinction of general life, had already been published by the author, in the year 11; since that epoch, he has considerably multiplied them, and corroborated the results which he formerly obtained. The following is the order in which the contractile organs of a healthy man, killed by decapitation, appeared to M. Nysten, to become insensible to the galvanic stimulus. First, the aortic ventricle; then, the intestines, stomach, bladder, pulmonary ventricle, œsophagus, iris, muscles of animal life; lastly, the auricles of the heart.

M. Nysten has thus demonstrated, by means of galvanism, what many authors had asserted before, the sensible organic contractility of the iris after death. He has also confirmed Bichat's observation upon the absence of this property in the arteries. From the different experiments which he has made upon various animals with red blood, it results that the excitability after death is less in proportion as the muscular energy has been more developed during life.

Examining the contractile power in subjects that had died from disease, he found it was affected more by the duration than the nature of the malady.

The work concludes with an inquiry into the stiffness of bodies after death, with the circumstances which influence its degree and duration. The author regards it as an unequivocal sign of death, and gives an exposition of the character by which it may be distinguished from tetanic stiffness and that of congelation.

Such are the principle facts contained in this book; they are neither new nor extraordinary, but they have some appearance of accuracy, are clearly narrated, and confirm the observations of previous inquirers; there is still, however, a considerable space to fill up between the maturity of Bichat, and the precocious labour of his emulous pupil, whose present performance can, with no sort of propriety, be regarded as a continuation of that of Bichat.

---

*Of the Medicinal Leech. By* M. Vitet, Professor in Medicine, Honorary Member of the Academy of Lyon, and of the Agricultural Society of the department of the Seine*.

We are informed in an advertisement by the editor, that M. Vitet was occupied 15 years in the composition of this work, and that in 1801 he caused the plate which accompanies it explanatory of his descriptions, and representing the most remarkable parts of the external and internal structure of the Leech, to be engraved; the editor, in consequence, asserts his priority over those modern authors who have published their works before him. It is, however, to be regretted that this work was not published in 1801, with the engraving, for we are now obliged to accord the rank of priority to M. Thomas, D. M. M. who published, in 1806, a treatise entitled, "*Memoire pour servir a l'histoire naturelle des sangsues.*"

Cuvier also would merit the precedency, as in his lectures on comparative anatomy, printed in 1800, there is a good anatomical description of the leech.

It is asserted in the advertisement, "that till the present time, the Leech was very imperfectly known, as one of the most useful remedies, and at the same time, one of those the most frequently applied in medicine; and that we had to correct

---

* Journal de Medicine.

many many false notions respecting its structure, functions, and effects." This supposition is completely refuted by the writings of the ancients; they made great use of the Leech, and regarded it as a very powerful remedy.

Themison, who lived in the first years of the Christian æra, used it on several occasions, and frequently applied cupping glasses to the part which the Leeches had quitted, to extract a greater quantity of blood.

Oribasius, towards the fourth century, wrote on the advantage of bleeding with Leeches. Aretæus recommended them in angina, accompanied with dyspnœa, when the patient feared the lancet.

Paulus Æginus, in the reign of Theodosius, applied them to the occiput, for pains of the head with fever, &c.

Ætius, in his book *de atra bile*, mentions his successful application of them upon the region of the liver in diseases of that organ. He also found them serviceable in tumours, diseases of the skin, the bites of venomous animals, and violent affections of the head, applied to the veins of the nose, the forehead, the temples, and behind the ears.

Horatius Augenius, in his treatise *de sanguinis missione*,* contemns bleeding children by means of the lancet, and advises Leeches to be substituted for it. Zacutus Lusitanus, in *de medicorum principum historia*, mentions a case in which he applied, at four different times, forty Leeches to a young man's face, which was affected with ulcerated pustules; they were healed, in consequence, in a few days. The same author also has related a curious history of a man who laboured under a sensation of heat in the back, and an unpleasant feeling of the skin, so insupportable, that he declared death would be preferable to the longer endurance of life with sufferings which had entirely deprived him of sleep. Zacutus finding that the remedies which had been employed were ineffectual, directed the patient to be placed for an hour every morning during the spring and summer, naked, in a brook frequented by Leeches. The patient pursued this plan for six months, at the end of which time he was cured.

Many other ancient authorities might be cited to prove that

---

* *Horatius Augenius*, a writer on medicine very little known, was born about 1527, and died at Padua in 1603, where he was professor of the theory of medicine. There remain now seven works of his on medical subjects; the one here cited has the title "de curandi ratione per sanguinis missionem, lib. xvii." The three first books were printed at Venice in 1570—reprinted with the remaining books, 4to. Venice 1597—folio, Francfurt, 1598. The author treats largely of the use of leeches and cupping, and of the methods of applying these remedies.

Editors.

 the the functions and medicinal effects of Leeches were known to physicians in the most remote ages.

M. Vitet could not have been ignorant that the anatomy of the leech had been ascertained by Redi, Vallisnieri, and Reaumur; that descriptions of it are given by Poupart in the *Journal des savans* for 1697; by Dillenius, in the *Ephemerides naturæ curiosæ*, in 1718; by Morand, in the *Mémoires de l'Académie des Sciences*, in 1739; by Durondeau, in the *Journal de Physique* de l'abbé Rosier, 1782; by Bibiéna, in the *Commentaires de l'Institut de Bologne*, 1791; by Cuvier, in his Lectures on Comparative Anatomy, in 1800; and lastly, by M. Thomas, in 1806.

The work of M. Vitet is divided into nine chapters. The first contains the anatomical description of the leech. In the second its functions are described. The third gives an account of the author's experiments upon the leech. The fourth treats of the sensible effects of the leech upon a person in health. The fifth, upon a person in disease. The sixth offers reflections upon the nature of blood extracted by leeches. In the seventh, the inconveniences attending the application of leeches are considered. The eighth, enumerates those diseases in which bleeding with leeches is indicated. The ninth chapter directs the mode in which they should be applied.

Chap. I.—*Anatomy of the Leech.*—M. Vitet has conducted his experiments upon the leech employed in medicine, *hirudo medicinalis*, Linn. to which he gives as characters of the species, *hirudo depressa nigricans, supra lineis flavis sex, intermediis nigro arcuatis, subtus cinerea nigro maculata, habitat in aquis dulcibus.* He begins with a description of the teguments, which he divides into epidermis, papillous coat, colored coat, and muscular coat. The epidermis is fixed, transparent, covers the whole surface of the body to which it adheres, and penetrates into all the external openings which it lines. When it is detached, a number of small holes or pores may be seen by the aid of a magnifying glass. The epidermis is readily separated from the colored coat in the dead leech, by putting it in vinegar or oil of turpentine.

Mr. Thomas thinks that the epidermis of the leech differs in structure from that of other animals, although it performs similar functions: he is induced to believe that it nearly resembles serous membranes.

The colored coat, situated between the epidermis and the papillous coat, viewed with a magnifying glass, presents the appearance of a spongy texture, in which is lodged the coloring matter. The papillous coat lies between the colored coat and the muscular coat. The papillæ differ in size and form: between each of them are cellular membrane, nerves, and blood vessels.

The

The muscular coat, situated beneath the papillous coat, incloses all the internal parts of the leech; it is of a dark grey colour, and is more or less thick, according to the different parts of the body. This coat has two sorts of fibres; one circular, having a spiral direction; the other, interior, is chiefly longitudinal. These are interlaced and united by fibres which prevent them from separating, even in the dead and macerated leech. These two sets of fibres change in direction round the external openings of the body; and interlace together, so as to form a kind of sphincter.

The external openings appertain to the head, the belly, and the back. The head has only one opening, which is the mouth: its edges are called the lips; they are divided into upper lip and lower lip. The external surface is of a dark grey, and the internal of a light grey colour. A number of small papillæ are on the surface, from which a bland and limpid fluid continually exudes in small quantity.

The openings in the belly are two in number; one anterior, named generative; the other posterior, called opening of the vagina.

The generative opening, round and small, is situated in the middle of the belly, about ten lines from the inferior lip of the mouth in a leech four inches long; it has a kind of muscular sheath, which has the power of enlarging, elongating, and retracting itself.

The opening of the vagina, situated in the middle of the belly, is smaller than the generative opening, from which it is distant two lines; it communicates immediately with the vagina, a short, narrow canal, having a delicate membrane continuous with the womb.

The openings of the back are two in number; one anterior, named dorsal; the other posterior, termed anus.

The dorsal opening, situated upon the brown band which occupies the middle of the back, is nearer the tail than the head: there proceeds from it a papilla, open in its centre, which supplies a serous matter. The author has not been able to discover the internal part with which this dorsal opening communicates, nor ascertain the use of the papilla observed upon it. M. Thomas has been more successful in his researches; he says, that it is by means of this membranous sac that the excretion of the glutinous humour, which restores the leech, is performed; and that if this humour, which lubricates the exterior surface of the skin, be absorbed either with powder or with paper, or fine linen, the animal makes efforts to expel a larger quantity of the fluid from the sac. He also contends, that the skin contains glands or follicles, which secrete the unctuous and glutinous fluid with which the leech is always covered.

The

The anus is situated towards the posterior extremity of the brown band of the back; it is continuous with the intestine, and is provided with a sphincter.

*Organs of digestion.* The teeth, three in number, have the form of an obtuse angle, with a cutting dentated edge; they rest upon small, whitish, fleshy masses, to which they adhere so tenaciously, that it is impossible to extract a tooth without bringing away with it some muscular fibres.

The author next demonstrates the œsophagus, its serous ducts, the alimentary canal, and the stomachs; the structure of the last is curious. They are twenty-six in number, and are situated regularly along the course of the alimentary canal, one below another, to the number of thirteen on each side; they are of a whitish colour, susceptible of contraction and relaxation; and all of them open into the alimentary canal.

The number of stomachs however is not always, as M. Vitet asserts, twenty-six. Out of a hundred leeches examined by M. Jacquemin, only one contained that number; two had twenty-five; and most of the rest twenty-four, as has been observed by Cuvier.

In each stomach may be distinguished the opening and the extremity. The opening towards the head is the widest part; the extremity much narrower, is directed to the posterior part of the tail: it is round, and fits into the next stomach behind it. Thus, divided from space to space by membranous diaphragms which streighten them, they join one into another.

This description of the alimentary canal certainly does not give a correct idea of its organization. An excellent demonstration of it may be seen in M. Cuvier's work.

*Neurology.* This comprehends the brain, the lateral ganglion, and the two great lateral nerves.

The brain is enclosed, with the organs of generation, in a sort of sac, situated between the posterior part of the œsophagus and the first stomach; it is divided into two equal portions, termed lobes, united by loose cellular membrane; it furnishes a nervous longitudinal cord, composed, according to this author, of twenty-eight ganglions, and, according to Cuvier, of twenty-three. Each of these ganglions supplies nervous filaments, which are distributed beneath the internal canal; they produce, on each side, two nerves which penetrate the substance of the longitudinal and transverse muscles.

M. Thomas has observed, that the nerves of leeches are nearly insensible to stimulants, whether mechanical or chemical. We may divide them partially, without the animal appearing to suffer, in the same manner as we may divide them wholly, without the neighbouring parts appearing to suffer much: this, doubtless, is owing to the numerous centres tres of action established by the ganglions, and to the isolation of their vitality. Sensibility in leeches presents a variety of modifications; this has been shewn by Batarra, who placed marine leeches in fresh water: they died in two hours—whilst salt water is fatal to common leeches.

*Angiology.* The blood-vessels of the leech are tubes nearly cylindrical, filled with red blood. The author thinks that they ought not to be divided into arterial and venous. All the means that he has tried to ascertain the motions of systole and diastole, have failed; he has never been able to observe them. M. Thomas, however, is of a different opinion; he says that he has seen, on each lateral part of the body, a membranous vessel full of red blood, extending from one extremity of the body to the other, having a regular systole and diastole, which occur very slowly; for only seven or eight pulsations are performed in a minute.

*Organs of generation.*—These are enclosed in the same membranous sac with the brain: they comprehend, 1. fœcundating vesicules, situated on the anterior part of the lobes of the brain; they are terminated by a canal which communicates with the generative conduit. 2. The generative conduit, which folding back twice on itself, gives rise to a filiform tube: this, slender, white, and nearly cylindrical, is about six or eight lines in length, out of its sheath. The living leech sometimes makes it project beyond the external opening; it is then more or less stiff and long, its extremity usually faces the tail. Occasionally a small drop of a limpid fluid may be observed at the exterior orifice of this conduit. 3. The womb; it is a kind of bladder, of an oval form, situated behind the two lobes of the brain, and terminates towards the left side, in the manner of a bag-pipe, by the vagina. The parietes of this organ are composed of a membranous and a muscular coat. 4. The ovary, which is a round white body, of a pretty firm consistence; it terminates by a slender cylindrical tube, which goes directly towards the middle of the anterior part of the womb, with which it communicates.

To Bibiena we owe the discovery of the mode of hermaphroditism in the leech; he was the first who spoke of it, without, it is true, having described the organs which constitute this species of hermaphrodite in a satisfactory manner, especially the organ which performs the function of the penis, a description of which may be found in the work of Cuvier, in that of Thomas, and of Vitet. Bibiena supposed that leeches possessed two modifications, or two species of hermaphroditism, that is, that the same individual being of both sexes, it might, by means of its organization, either impregnate itself without the union of another individual, or it it might be impregnated by reciprocal copulation. He says, *Primum itaque conjicio, hermaphroditum hunc nostrum in illis esse numerandum, qui et cum aliis suæ speciei copulari possunt, et sibimet ipsis etiam conjungi, patris simul matris officio fungentes.*

M. Thomas, considering particularly the length of the penis, determined, in some measure, by the distance between the opening of the vagina and that of the male organs, thinks that reciprocal copulation can only be performed by the two individuals presenting themselves to each other, in an opposite direction. His observations have enabled him to determine with Rédi, Durondeau, and others, that the leech is viviporous; the young ones are like threads; it is only by a progressive growth that they acquire the form peculiar to the leech.

When the leech is disposed to multiply itself, says M. Vitet, it curves back on itself; the generative tube issues from its sheath, it stiffens, elongates, and penetrates into the vagina, situated about two lines below the opening of the generative member. The author exerts himself ineffectually to explain what passes during this mysterious act, whether in what concerns the seminal emission from the male organ, or in describing the contracting of the womb, at the moment of supplying the liquor for fecundation.

*Respiration.*—All the attempts of M. Vitet to discover the organs of respiration in the leech, by dissections, by injections, by the magnifying glass, or by the microscope, completely failed. He admits, however, that the leech respires, and that it seeks the external air, although water is its most frequent receptacle. Those which he subjected to the action of the pneumatic machine, gave out air-bubbles by the mouth, and surface of the skin; they died the sixth day of complete privation of the external air. M. Vitet, therefore, denies the opinion of authors who have asserted that the leech respires, by means of organs subservient to that function. All naturalists of the present day agree that the leech respires; the experiments of M. Thomas leave no doubt on the subject. He says, that at certain distances on each side of the body of the leech, there are transparent membranous sacs, of a vesicular form; they appear to contain only air, and are placed in a regular manner on both sides the animal. Numerous blood-vessels ramify on these respiratory organs, and the air passes to them through follicles on the surface of the skin.

*Of the Senses.*—The senses of the leech relate chiefly to feeling, which resides principally in the internal and external surface of the lips.

The

The smell is a kind of feeling, by which the Leech ascertains the odorous substances that are noxious or useful to it.

Taste is the sense which enables the Leech to discern the savour of the blood, and which causes it to prefer this fluid to every other nutritive substance of which animals make use. Milk, sugar and water, yolk of eggs, &c. are nutritive fluids for which the Leech shews more aversion than desire. It is needless, then, says the author, to bathe with milk, or sugar and water, the part of the integuments where you wish the leech to fasten; instead of these substances, he proposes that the skin be well washed with warm water, or rubbed till it is red, when the Leech will bite with the greatest promptitude.

*Experiments with Leeches in water and out of water.*—The Leech kept in a bottle with free access to the air, and the water frequently changed, will live six years. Kept in a vessel without water, far from acquiring in the first six or eight hours vigour and eagerness to bite, it loses all power. It is not advantageous, therefore, to keep it dry in a vessel, with the expectation of its biting more readily.

Leeches never bite each other; they often lye upon one another in a heap, but seem rather to caress than to wound each other.

The Leech will live a long time in water without either mouth or tail.

The quantity of blood which a strong Leech can draw, never exceeds an ounce. M. Vitet then gives a description of various experiments which he has made with different oils, camphor, alkalis, metallic salts, gases, the electrical machine, and galvanic pile, to ascertain the sensibility and vital force of the Leech.

Exposed to the action of the galvanic pile, the Leech contracted and bent back on itself, the mouth disappeared, and the extremity of the tail was drawn in. As soon as it was put into water it resumed its former state. Similar effects took place on the application of electricity.

In the 4th chapter, the author says, that Leeches do not act alike, on the infant, the young man, the adult, the old man, or the female. They fasten on the vessels of infants with avidity; they suck much blood, and when they drop off, the wounds bleed freely. This is not the case with the other individuals.

In the 5th, 6th, and 7th chapters, M. Vitet opposes the practice of bleeding with the lancet: he gives a decided preference to Leeches, in all inflammatory diseases. In pressing cases, however, such as comatose affections, wounds with great disturbance of the sensorium, sudden suppression of habitual sanguineous evacuations, followed by urgent symptoms, he recommends the lancet. When Leeches are used, he generally prefers applying them to the thighs.

The 8th chapter treats of the diseases in which Leeches are indicated: it contains little that is new or interesting.

In the 9th chapter, the author teaches the art of applying Leeches, and the means of stopping the flow of blood when they have fallen off. This chapter is beneath his reputation, and had better have been omitted.

Upon the whole, much curious matter is contained in this work; from the method which he has pursued, the great research which he has evinced, and the minute anatomical description which he has given of the Leech, M. Vitet has performed a difficult task, and merits a distinguished place amongst the authors who have written on this branch of natural history.

